# Dissecting nutrient-related co-expression networks in phosphate starved poplars

**DOI:** 10.1371/journal.pone.0171958

**Published:** 2017-02-21

**Authors:** Mareike Kavka, Andrea Polle

**Affiliations:** 1 Forstbotanik und Baumphysiologie, Georg-August Universität Göttingen, Göttingen, Germany; 2 Labor für Radio-Isotope, Georg-August Universität Göttingen, Göttingen, Germany; INRA, FRANCE

## Abstract

Phosphorus (P) is an essential plant nutrient, but its availability is often limited in soil. Here, we studied changes in the transcriptome and in nutrient element concentrations in leaves and roots of poplars (*Populus* × *canescens*) in response to P deficiency. P starvation resulted in decreased concentrations of S and major cations (K, Mg, Ca), in increased concentrations of N, Zn and Al, while C, Fe and Mn were only little affected. In roots and leaves >4,000 and >9,000 genes were differently expressed upon P starvation. These genes clustered in eleven co-expression modules of which seven were correlated with distinct elements in the plant tissues. One module (4.7% of all differentially expressed genes) was strongly correlated with changes in the P concentration in the plant. In this module the GO term “response to P starvation” was enriched with phosphoenolpyruvate carboxylase kinases, phosphatases and pyrophosphatases as well as regulatory domains such as SPX, but no phosphate transporters. The P-related module was also enriched in genes of the functional category “galactolipid synthesis”. Galactolipids substitute phospholipids in membranes under P limitation. Two modules, one correlated with C and N and the other with biomass, S and Mg, were connected with the P-related module by co-expression. In these modules GO terms indicating “DNA modification” and “cell division” as well as “defense” and “RNA modification” and “signaling” were enriched; they contained phosphate transporters. Bark storage proteins were among the most strongly upregulated genes in the growth-related module suggesting that N, which could not be used for growth, accumulated in typical storage compounds. In conclusion, weighted gene coexpression network analysis revealed a hierarchical structure of gene clusters, which separated phosphate starvation responses correlated with P tissue concentrations from other gene modules, which most likely represented transcriptional adjustments related to down-stream nutritional changes and stress.

## Introduction

Phosphorus (P) is an important plant nutrient and essential for plant growth and metabolism. But P is also one of the least available nutrients in soil [1]. Plants take up P only in its inorganic form, phosphate [2]. Free phosphate is present in very low concentrations in the soil solution (< 10 μM; [3]) because it is bound to soil particles, to organic compounds, and forms precipitates with other soil elements like iron (Fe), aluminum (Al) and calcium (Ca) [1].

To acquire and translocate phosphate, plants possess a number of different phosphate transporters (PHTs). In *Arabidopsis thaliana*, plasma membrane located PHTs of family 1 are responsible for P uptake into the root and show strong up-regulation under P deprivation [4,5]. In poplar, the expression of orthologs of the *AtPHT1* genes is also strongly increased upon P starvation [6–9]. Furthermore, the expression of distinct purple acid phosphatases is enhanced under P deficiency [6]. Phosphatases and organic acids, which are secreted by the roots, increase phosphate availability in soil and together with enhanced P uptake capacities, P acquisition is increased under P starvation [7,10]. Altogether adaptation to limiting P supply results in reduced growth, but higher P use efficiency [6,7].

P deficiency affects not only P uptake and utilization, but also other plant nutrients. For example, in *Arabidopsis* P starvation caused decreased potassium (K) and enhanced iron (Fe) concentration in the leaves [11–13]. Furthermore, reductions in calcium (Ca), magnesium (Mg) and manganese (Mn) were detected; it was speculated that these changes may indicate an adjustment of the ionic charge [11,13] or could be the result of reduced P availability in common P requiring steps of metabolism and transport [12]. There are obviously interrelations among processes that regulate plant nutrient balance because deprivation of tomato in either P or K or Fe resulted in rapid and partly overlapping transcriptional responses [14]. In *Arabidopsis*, a number of genes, which are highly regulated in response to P starvation [12,15–17], are also required for the utilization of other elements, e.g., *SULTR1;3* for sulfate transport and *NAS1* for iron chelation by nicotianamine [18,19]. In poplar, reduced P availability leads to higher nitrogen (N) concentrations in roots and lower N concentrations in leaves with higher free amino acid concentrations and reduced activities of N-related enzymes (nitrate reductase (NR), glutamine oxoglutarate aminotransferase (GOGAT), glutamine dehydrogenase (GDH); [6,7]). However, comprehensive analyses how P deficiencies affect the tissue concentrations of essential nutrient elements in poplar and whether these changes are related to alterations in the transcriptome are lacking.

The main goal of this study was to characterize changes in nutrient element concentrations (P, S, C, N, K, Mg, Ca, Fe, Zn, Mn, and the non-essential element Al) in response to P starvation in poplar (*Populus* × *canescens*) and to differentiate between transcriptomic changes directly correlated with the plant P concentrations and transcriptomic changes linked to down-stream changes of other nutrient concentrations. For this purpose, we studied poplar transcriptomes under high, intermediate and low P availabilities in leaves and roots, determined weighted gene co-expression networks and identified gene modules that were correlated with nutrient element concentrations. The network showed a hierarchical structure with a top module related to P concentrations that was connected with two main secondary modules, one related to C and N and the other to Mg, S and biomass. The secondary modules were connected by co-expression to tertiary modules. The identified gene modules and their putative functions in the poplar P starvation response are discussed. As the consequence of P starvation the tissue concentrations of other nutrient elements also show massive modification. A significant outcome of our study is that the primary P starvation responses can be disentangled from down-stream gene regulation related to changes in other nutrient elements.

## Materials and methods

### Plant material, growth conditions and harvest

Growth of *Populus* × *canescens* (INRA717 1-B4) with three different P availabilities has been reported before [7]. Briefly, the plants were grown in sand culture and irrigated with one of three nutrient solutions. Long Ashton nutrient solution [20] with 641 μM P was used for control plants (high phosphate–HP). For intermediate phosphate (MP) availability, the phosphate concentration was reduced to 6.41 μM and potassium added as KCl (675.8 μM), for low phosphate (LP) availability to 0.0641 μM (additional 682.5 μM KCl).

After two months of growth with different P supplies, the poplars were harvested. Aliquots of the plant tissues (leaves, stem, coarse roots, fine roots < 2 mm diameter) were dried at 60°C for seven days for determination of biomass (n = 10 per treatment): $\text{tissue}\;\text{biomass}\;[\text{g}]=\frac{\text{dry mass of aliquot}\;[\text{g}]\times \text{total tissue fresh mass}\;[\text{g}]}{\text{fresh mass of aliquot}}$.

The first three leaves from the top (> 2 cm length) and aliquots of fine roots (< 2 mm diameter) were immediately shock frozen in liquid nitrogen and stored at -80°C for RNA-extraction.

### Element concentrations

Dry tissues of 2-month-old HP-, MP- and LP-poplars (n = 4 per treatment) were milled (Retsch, type MM2, Haan, Germany) and 10 to 45 mg of plant powder was pressure-extracted in HNO_3_ [21]. Element concentrations were measured using an inductively coupled plasma optical emission spectrometer (ICP-OES; Optima 5300 DV, PerkinElmer Life and Analytical Sciences, Rodgau, Germany). For determination of carbon and nitrogen concentrations, 0.7 to 0.9 mg dry plant powder (2-month-old HP-, MP- and LP-poplars, n = 5 per treatment) was weighed into tin capsules (Hekatech, Wegberg, Germany) and analyzed in duplicates in an Elemental Analyzer EA1108 (Carlo Erba Strumentazione, Rodano, Italy). Acetanilide (71.09% C, 10.36% N; Carlo Erba Strumentazione) was used as the standard.

### RNA-extraction and microarray

RNA-extraction and microarray procedures at the Microarray Facility (MFT Services, Tübingen, Germany) were described by Kavka and Polle [7]. Three biological replicates (each consisting of two pooled tissue samples) were analyzed per treatment and tissue (fine roots and uppermost leaves).

### Statistical analyses of microarrays

Microarray raw data were analyzed using the free statistic software R (version 2.14.2; [22]) after the protocol described by Janz et al. [23]. The R package “affy”[24] was used for normalization of the array probes (“rma” function) using Bioconductor [25]. The log_2_ expression value of transcripts that were present (“mas5calls” function) on all replicate chips of at least one condition was used for further analyses. For the annotation of the microarrays, the best gene model for each Affymetrix ID (AffyID) in the annotation file of the Aspen Database was used [26]. Differentially expressed genes (DEGs) were identified by two methods: (a) Significance Analysis of Microarrays (SAM) was performed with R package “siggenes” [27] (FDR ≤ 0.05) and (b) the data were analyzed using linear models with R package “limma” [28] (adjusted p-value ≤ 0.05). Overlapping DEGs identified by both approaches were used for further analyses. The microarrays have previously been validated by quantitative real time polymerase chain reaction of phosphate transporter expression data [7]. Venn diagrams of DEGs were drawn using the online tool InteractiVenn (http://www.interactivenn.net/; [29]). Log-fold change ratios (logFCs) were calculated with the mean expression values for MP/HP and LP/HP for roots and leaves.

A weighted correlation network analysis with all DEGs was conducted using R package “WGCNA” (Weighted Gene Co-expression Network Analysis; [30]). Block-wise clustering with a power of 24 was calculated and modules in the resulting dendrogram were merged at a height cut of 0.2. Pearson’s correlations of eigengenes, which represent the expression profile of all genes in a given co-expression module, and mean values of element concentrations and biomass in fine roots and leaves of HP, MP and LP plants were calculated. Because the large number of DEGs did not allow a visualization of the co-expression network with the usual tools, gene numbers were reduced by using a p-value (limma) cut-off of 0.00001. Topology was recalculated for these genes by using the same power of 24 (command “TOMsimilarityFromExpr”). Nodes (DEGs) and edges (co-expression connections between nodes, based on expression pattern) were displayed in Cytoscape (version 3.3.0; [31]) in group attributes layout to show DEGs belonging to one module.

GO term enrichment analyses were conducted using Ontologizer (version2.1; [32]) with Term-for-term approach and Benjamini-Hochberg-corrections. Enrichment in poplar gene lists were tested with p ≤ 0.01 against a “population” data set consisting of all genes present on the array as described above. Enriched GO terms were displayed using GOPathDrawer (version 6.0, http://coxpresdb.jp/top_draw.shtml). For an overview, significant GO terms were uploaded into REVIGO (http://revigo.irb.hr/, accessed October 2016; [33]), which used the GO term version Jul 2015 monthly release "go_201507-termdb.obo-xml.gz" and plotted as TreeMap (setting of similarities: 0.9).

### Additional statistical analyses

ANOVA and Tukey’s HSD were performed in R (version 2.14.2; [22]) to test for differences between treatments in biomass and element concentrations with p ≤ 0.05. Residuals were tested visually for normal distribution and homogeneity of variance and data transformed logarithmically (log_2_), by square root or by the exponent “2” if needed. Differences between treatments at p ≤ 0.05 are shown with different letters in figures. Correlations of mean values were calculated using Pearson's test in R.

### Data availability

Raw and normalized data of the microarrays were uploaded into the EMBL-EBI ArrayExpress database (http://www.ebi.ac.uk/arrayexpress/) under E-MTAB-3934. Data for biomass, nutrient elements concentrations and count tables with information on the transcript abundances of all genes are available from the Dryad Digital Repository: http://dx.doi.org/10.5061/dryad.3t5q5.

## Results

### P deprivation affects nutrient element concentrations

**Fig 1:**
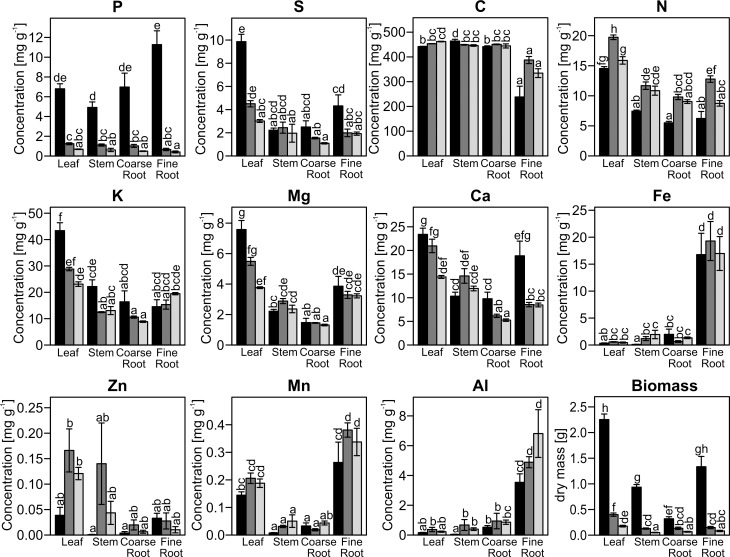
Element concentrations in leaves, stem, coarse roots and fine roots of poplars. *P*. × *canescens* was grown with high (HP, 641 μM, black), intermediate (MP, 6.4 μM, dark grey) or low (LP, 0.064 μM, light grey) P supply. Different letters indicate significant differences (p ≤ 0.05, Two-Way-ANOVA and Tukey's honest significance test, mean±SE, n = 4–10. P concentration and biomass were taken from Kavka and Polle [7]. S: sulfur, C: carbon, N: nitrogen, K: potassium, Mg: magnesium, Ca: calcium, Fe: iron, Zn: zinc, Mn: manganese, Al: aluminum.

The concentrations of sulfur, nitrogen, potassium, magnesium, calcium, iron, zinc, manganese and aluminum were influenced by P starvation, but the differences were generally smaller than those for P (Fig 1, Table 1). Sulfur, magnesium and calcium concentrations declined in all tissues except the stem in response to MP or LP treatments (Fig 1, Table 1). Potassium was slightly reduced in all tissues except in fine roots, whereas iron, manganese, zinc and aluminum concentrations increased in most tissues in response to P deficiency (Fig 1, Table 1). Nitrogen concentrations were higher in MP than in HP poplars in all tissues. The carbon concentration did not change markedly under low P availability (Fig 1, Table 1).

**Table 1:** ANOVA results for the main factors “Treatment” and “Tissue”.

Parameter	Treatment	Tissue	Interaction
P	**<0.001**	0.225	**0.003**
S	**<0.001**	**<0.001**	0.116
C	0.641	**<0.001**	**<0.001**
N	**<0.001**	**<0.001**	**<0.001**
K	**<0.001**	**<0.001**	**0.002**
Mg	**0.007**	**<0.001**	**0.007**
Ca	**<0.001**	**<0.001**	**<0.001**
Fe	**0.001**	**<0.001**	**<0.001**
Zn	**0.026**	**<0.001**	0.094
Mn	**0.020**	**<0.001**	0.593
Al	**0.001**	**<0.001**	0.074
BM	**<0.001**	**<0.001**	**<0.001**

P-values of linear models (Two-Way-ANOVA) were calculated for element concentrations and biomass (BM) (see Fig 1) with the factors treatment (HP, MP, LP) and tissue (leaves, stem, coarse, fine roots) and a factor of interaction. Significant treatment effects and interactions with p ≤ 0.05 are indicated in bold.

Although the changes in element tissue concentrations were caused by differences in P availability in the growth medium, they did not correlate with the P concentrations in the different tissues (Table 2). For other nutrient elements, we detected three groups showing correlations: (i) S, K, Mg, and S with positive relationships, (ii) N, Mg, and Zn with positive relationships and (iii) C, Fe, Mn, and Al, where the latter three were positively related to each other and negative with C (Table 2). Furthermore, some elements were correlated with biomass (P: p = 0.005, r = 0.748, S: p<0.001, r = 0.884; K: p = 0.007, r = 0.731; Mg: p = 0.010, r = 0.706; Ca: p = 0.011, r = 0.700).

**Table 2:** Correlation analysis of element concentrations.

	P	S	C	N	K	Mg	Ca	Fe	Zn	Mn	Al
P		0.505	-0.504	-0.443	0.266	0.257	0.465	0.147	-0.290	-0.008	-0.055
S	0.094		-0.052	0.393	**0.885**	**0.911**	**0.855**	-0.136	0.191	0.148	-0.213
C	0.095	0.873		0.397	0.163	-0.103	-0.128	**-0.842**	0.260	**-0.637**	**-0.742**
N	0.150	0.206	0.201		0.534	**0.643**	0.519	-0.260	**0.779**	0.256	-0.240
K	0.404	**0.000**	0.614	0.074		**0.888**	**0.743**	-0.207	0.231	0.195	-0.201
Mg	0.420	**0.000**	0.751	**0.024**	**0.000**		**0.881**	0.029	0.417	0.424	-0.022
Ca	0.128	**0.000**	0.692	0.084	**0.006**	**0.000**		-0.124	0.558	0.188	-0.252
Fe	0.648	0.673	**0.001**	0.415	0.518	0.928	0.701		-0.297	**0.838**	**0.939**
Zn	0.361	0.553	0.414	**0.003**	0.470	0.178	0.060	0.349		0.069	-0.309
Mn	0.979	0.647	**0.026**	0.422	0.544	0.169	0.559	**0.001**	0.832		**0.804**
Al	0.866	0.505	**0.006**	0.453	0.531	0.947	0.429	**0.000**	0.328	**0.002**	

Data for concentrations in leaves, stem, coarse and fine roots (see Fig 1) were used and p-values (lower diagonal) and the Pearson’s correlation coefficient r (upper diagonal) of correlation are shown. Correlations with p ≤ 0.05 are indicated in bold.

### P deprivation leads to massive transcriptional reprograming

**Fig 2:**
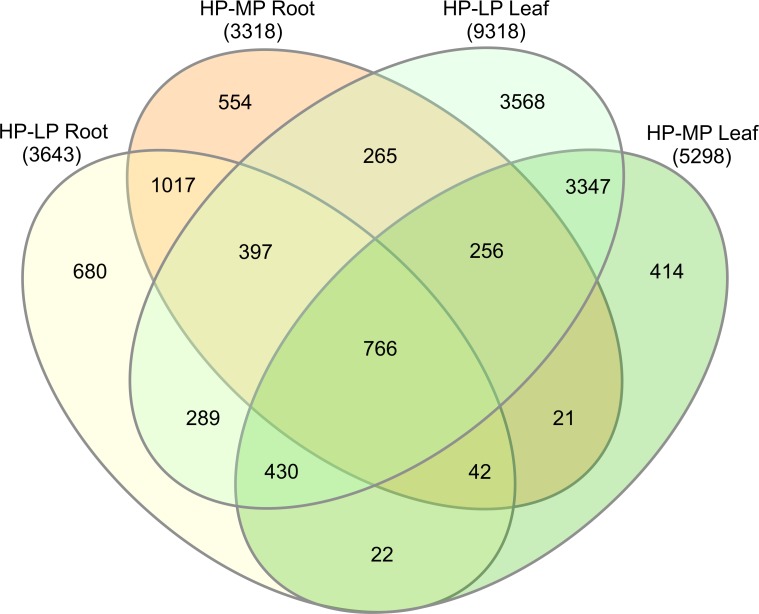
Venn diagram of differentially expressed genes (DEGs). *P*. × *canescens* was grown with high (HP, 641 μM), intermediate (MP, 6.4 μM) or low (LP, 0.064 μM) P supply. The numbers of DEGs between HP and LP and HP and MP in roots and leaves are shown.

A total number of 12068 genes were differentially expressed in response to MP or LP treatment compared with HP (Fig 2). Common to all P treatments were 766 DEGs. In general, roots contained less DEGs than leaves although the decline in P was more pronounced in roots than in leaves (Figs 1 and 2). There were 1017 root-specific and 3347 leaf-specific DEGs in response to both MP and LP treatments (Fig 2). The complete list of DEGs, their annotation and fold-changes are shown in S1 Table.

### Co-expression modules are correlated with nutrient element concentrations and biomass

**Fig 3:**
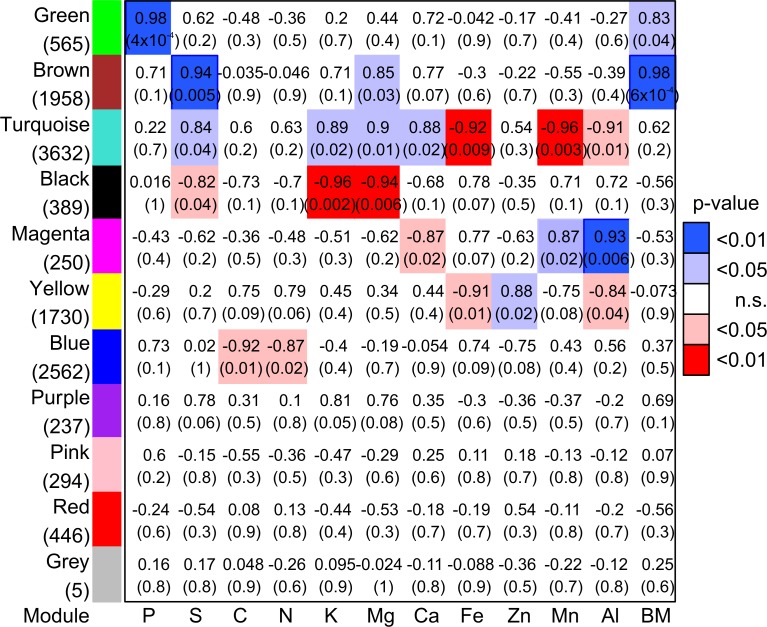
Correlation matrix between eigengenes of co-expression modules and element concentrations and biomass. Modules were composed of genes differentially expressed between poplars (*P*. × *canescens*) grown with high P availability and plants grown with intermediate or low P availability in fine roots or leaves. Correlation coefficient and p-value (in brackets) are shown. Significant p-values (≤ 0.05) of correlation are color coded (red: negative correlation, blue: positive correlation). Numbers of DEGs in modules are shown below module’s name.

To identify genes linked to P nutrition, co-expression modules were calculated and the eigengenes were related to the element concentrations in roots and leaves and the biomass of these tissues. The co-expression analysis resulted in eleven modules (named after colors), which contained 5 to 3632 DEGs (Fig 3, S1 Table). The eigengenes of four modules (“Purple”, “Pink”, “Red”, “Grey”) were unrelated to any one of the examined elements or biomass (Fig 3).

Eigengenes of module “Green” were strongly correlated with tissue P concentrations (p-value: 0.0004, r = 0.98). Module “Green” was also weakly correlated with biomass (p-value = 0.04, r = 0.83, Fig 3). Module “Brown” was positively related to sulfur (p = 0.0005, r = 0.94), magnesium (p = 0.03, r = 0.85) and biomass (p = 0.0006, r = 0.98). Eigengenes of module “Blue” were negatively correlated with carbon and nitrogen concentrations (Fig 3). The largest module “Turquoise” (3632 genes) showed positive correlations with sulfur, potassium, magnesium and calcium and negative correlations with iron, manganese and aluminum concentrations (Fig 3). Module “Black” showed negative correlations with potassium, manganese and sulfur concentration, and “Magenta” positive correlations with manganese and aluminum and a negative correlation with calcium (Fig 3). Module “Yellow” was negatively correlated with iron and aluminum, and positively correlated with the zinc concentrations.

Our analyses also revealed significant regulation of putative transporters with more than 100 annotated DEGs (S1 Table). The transport-related DEGs were spread across different modules without clear patterns related to element abundances.

### Network analysis reveals a hierarchy of modules

**Fig 4:**
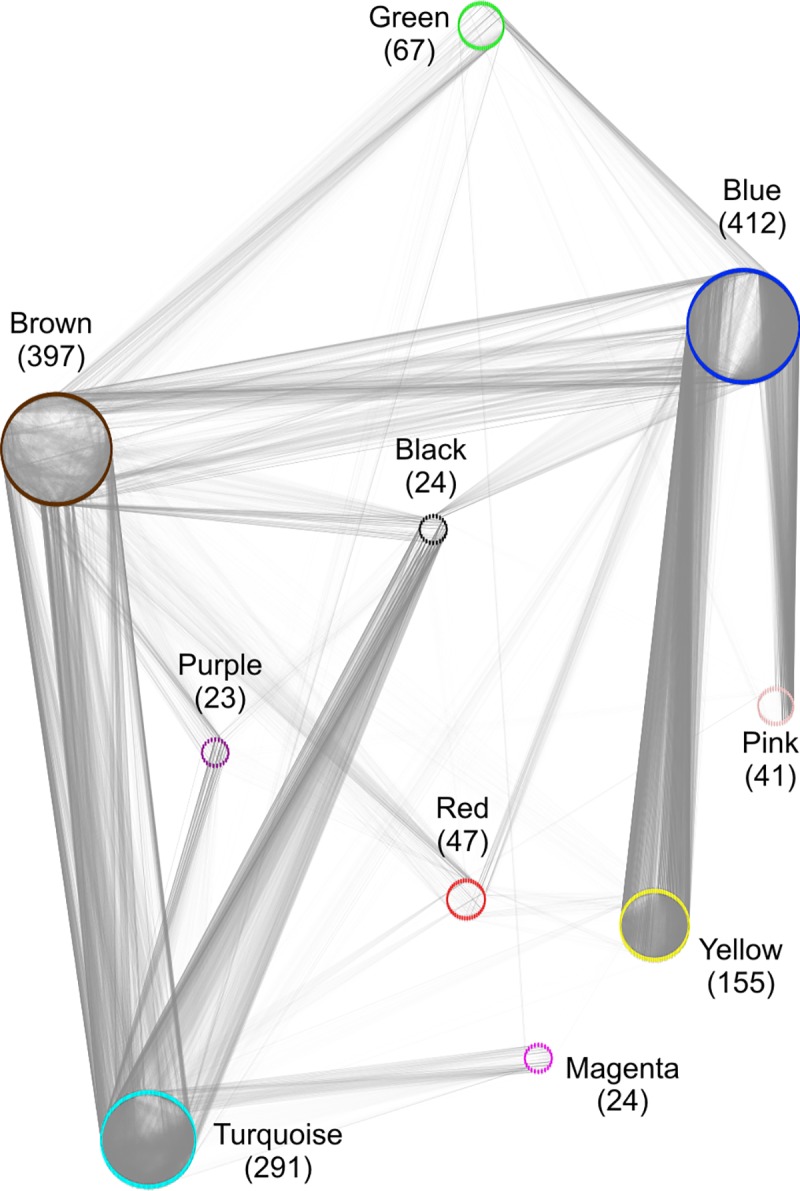
Co-expression within and between modules of DEGs. Due to computing power (about 12,000 nodes (= DEGs); about 45,000 edges (= co-expression between two DEGs) merely for module “Green”), only DEGs with p ≤ 0.00001 (limma) were drawn. Using a fold-change cut-off resulted in a similar network picture (not shown). Nodes (= DEGs of modules) are shown by their colors. Co-expression (Weighted Gene Co-expression Network Analysis) between two nodes is represented by grey line (edge). Higher adjacency between two DEGs is indicated by darker line color (adjacency threshold for edge drawing: 0.25). The reduced number of DEGs used to draw the network is indicated below module’s name.

Network analysis showed that module “Green”, which was strongly correlated with P concentrations, was mainly connected with “Brown” and “Blue” by co-expression (Fig 4). Links of “Green” with other modules were rare (“Magenta”, “Pink” and “Turquoise”) or did not exist (“Red”, “Black”, “Purple” and “Yellow”). Strong co-expressions were found between genes in module “Blue” with those in modules “Yellow”, “Pink”, and “Brown” (Fig 4). Module “Brown” was further strongly connected to module “Turquoise”, whose genes were co-expressed with genes in modules “Purple”, “Magenta” and “Black”. The network structure, thus, indicated co-expression of P-related genes with the C- and N-related genes (“Blue”) on the one hand and the S-, Mg- and biomass-related genes on the other hand (“Brown”, Fig 3, Fig 4). All other modules were apparently not strongly connected with the P-related module, and therefore, the elements correlated with these modules were neither strongly connected with changes in P concentrations.

### Functional characterization of P-related and highly connected modules

**Fig 5:**
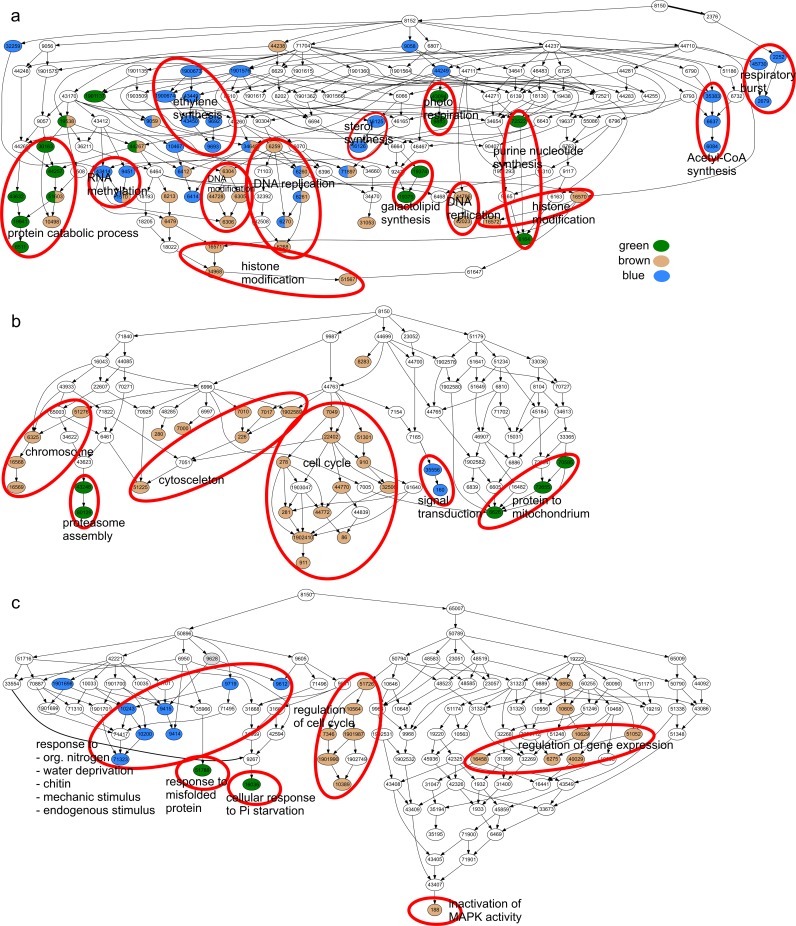
Hierarchy of enriched GO-terms of Biological Process in modules “Green”, “Blue” and “Brown”. Enriched GO terms are colored with module color, white: GO terms not enriched in modules. GO term hierarchy was drawn with GOPathDrawer. To enable presentation of all GO terms, the figure was split in three parts a), b) and c) and the connections between these parts were omitted.

**Fig 6:**
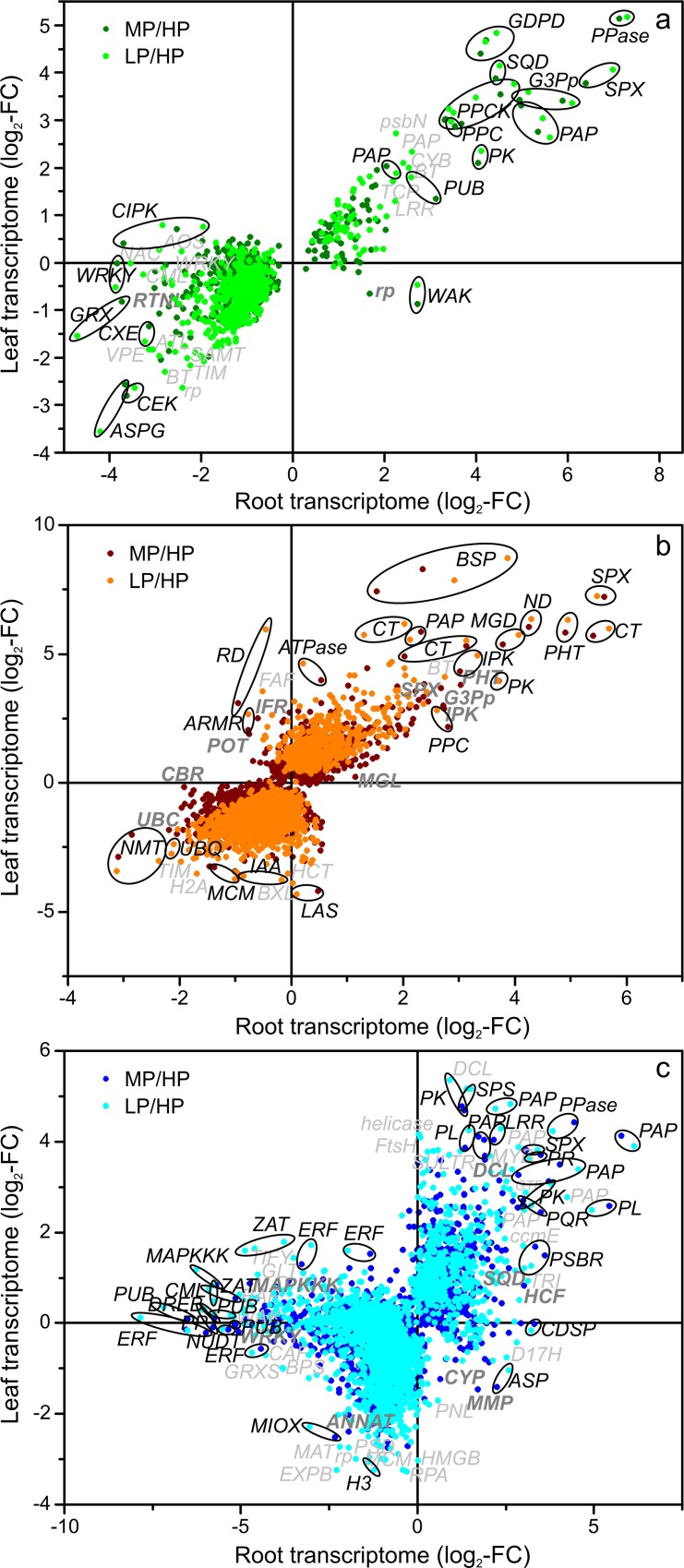
**Change in transcript abundance of P-responsive genes in roots relative to that in leaves for the modules “Green” (a), “Brown” (b) and “Blue” (c).** Log_2_-fold change of MP/HP and LP/HP DEGs in roots were plotted to the corresponding log_2_-fold changes in leaves. Abbreviations of the genes names are listed in S3 Table.

To characterize the modules functionally, GO term enrichment analysis was performed for all modules (S2 Table, S1 Fig) and is presented here for module “Green” and the two most strongly connected modules “Blue” and “Brown” (Fig 5). GO terms enriched in module “Green” were related to protein catabolism with terms “protein catabolic process”, “proteasome assembly” and “response to misfolded protein”. Further enriched GO terms in module “Green” were related to energy metabolism with “protein localization to mitochondrium”, “purine nucleotide synthesis” and “photorespiration” as well as to “galactolipid synthesis” and “cellular response to phosphate starvation” (Fig 5). The latter GO term encompassed two phosphoenolpyruvate-carboxylase kinases (Potri.013G046100, Potri.019G018100), a sulfolipid synthase (Potri.016G112600), an SPX gene (Potri.006G069500), an inorganic pyrophosphatase (Potri.003G034600) and a purple acid phosphatase (Potri.005G233400) (S2 Table, Fig 6A). Generally, genes in module “Green” showed similar responses to P starvation in roots and leaves (Fig 6A). The genes in GO term “cellular response to phosphate starvation” belonged to those with the strongest P-starvation induced up-regulation in the module “Green” in both roots and leaves (Fig 6A). For instance, glycerol-3-P transporters (Potri.003G109300, Potri.001G124200), the purple acid phosphatase Potri.010G158200, a phosphoenolpyruvate-carboxylase kinase (Potri.013G046100), a phosphoenolpyruvate-carboxylase (Potri.008G114200), a serine/threonine-protein kinase (Potri.006G109600) and a glycerophosphodiester phosphodiesterase (Potri.001G325200) were highly upregulated in roots and leaves. In module “Green” asparaginase (Potri.014G022900) and choline/ethanolamine kinase (Potri.005G197500) were strongly downregulated in roots and leaves upon P starvation. CBL-interacting serine/threonine-protein kinases (Potri.006G062800, Potri.019G128100) and WRKY-transcription factors (Potri.001G044500, Potri.018G019800) were downregulated only in roots of the P-starved plants. Wall-associated receptor kinase (Potri.002G075900) and a 50S ribosomal protein (Potri.005G154300) were downregulated in leaves but upregulated in roots (Fig 6A).

In addition to “Biological Process” we analyzed the enrichment of GO terms for “Molecular Function” and “Cellular Component” in the co-expression modules. In agreement with “Biological Process” we found GO terms related to the respiratory chain and mitochondrium enriched in module “Green” (S2 Table).

The P-related module “Green” was connected by 17268 edges with the biomass/S/Mg related module “Brown” (1958 DEGs) (cf. Fig 4). GO terms enriched in module “Brown” were related to growth (“DNA replication”, “cytoskeleton”, “cell cycle” and “regulation of cell cycle”), and to the regulation of gene expression (“DNA”and “histone modification”, “chromosome” and “regulation of gene expression”) (Fig 5). The expression of genes encoding an *SPX-domain* containing protein (Potri.014G061200), the phosphate transporter *PtPHT1;12* (Potri.001G318500), a chitinase (Potri.004G182000) and an NADH-dehydrogenase (Potri.011G044600) were especially highly upregulated in module “Brown” (Fig 6B). Transcript abundances for bark storage proteins (Potri.013G101000, Potri.013G100700) were among the highest upregulated genes in leaves (Fig 6B). Phosphoethanolamine N-methyltransferases (Potri.015G039000, Potri.012G047400) were strongly downregulated in both roots and leaves in module “Brown” (Fig 6B).

Genes of module “Blue” (2562 DEGs and 5330 edges with module “Green”) (cf. Fig 4) were enriched in GO terms related to regulation and signaling (“RNA methylation”, “signal transduction”, “ethylene synthesis”), and to general stress responses (“respiratory burst”, response to some stresses and stimuli e.g. “response to endogenous stimulus”) (Fig 5). Furthermore, GO terms enriched in genes of module “Blue” might be related to membrane lipid degradation (“sterol synthesis”and “acetyl-CoA synthesis”). Notably, among the genes of module “Blue” (Fig 6C) purple acid phosphatases (Potri.003G030700, Potri.005G233400, Potri.010G158200, Potri.015G031400, Potri.012G042200) were highly upregulated and ethylene-responsive transcription factors were mostly upregulated in leaves and downregulated in roots.

The comparison of the most strongly up- or downregulated DEGs in poplar with a similar study in *Arabidopsis* [12] revealed a huge overlap of the identified P-responsive orthologs (S3 Table).

## Discussion

### Regulation of P responses

Here, we present the transcriptional profiles of poplar roots and leaves in response to phosphate starvation. Leaves of P-starved poplars showed more DEGs than roots. This finding agrees with the P starvation response in *Arabidopsis* [34] and suggests that the response in leaves is more complex than in roots. Among 12068 DEGs, we identified one gene cluster (module “Green”) with 565 genes, which was correlated with the tissue P concentrations. Whether cause-effect-relationships exist between DEGs in module “Green” and the tissue P concentration is unknown, but the module contains orthologs of *Arabidopsis* transcription factors known to regulate P sensing and signaling [35,36]. It is, therefore, likely that this module is involved in the surveillance of the P status of the cell and in crucial metabolic adjustments because it was also enriched in functions indicating enhanced degradation activities, which may be required to achieve higher P recycling in the plant tissues.

Among the DEGs in roots and leaves several members of the SPX transcription factor family (highly expressed: Potri.006G069500, Potri.006G253400, moderately enhanced: Potri.014G061200, Potri.018G028200, Potri.006G069500) were detected, which are key regulatory factors of P homeostasis in plants [35,36]. It has already been demonstrated for several plant species that the SPX protein interacts with PHR (PHOSPHATE STARVATION RESPONSE REGULATOR), e.g., AtPHR1 in *Arabidopsis*, OsPHR2 in rice [37,38]. PHR enables the transcription of P responsive genes by binding to the P1BS element in the upstream region of those genes [37,38]. *Arabidopsis* mutants with suppressed PHR1 or a homolog transcription factor PHL did not recruit genes enriched in P1BS elements any more while its overexpression resulted in a P starvation response in the presence of sufficient phosphate [39]. SPX modulate the PHR responses: under high P conditions, SPX proteins bind PHR and thereby prevent the activation of phosphate responsive genes in a P dependent manner [37,38]. Up-regulation of the expression of SPX genes under low P conditions allows plants to react rapidly to P resupply because the binding of PHR stops the enhanced expression of PHR target genes [37,38]. Because the poplar SPX genes in module “Green” showed P concentration related transcript abundances, it is likely that they fulfill similar functions as their homologs in *Arabidopsis* and rice [37,38], where they link P perception and signaling.

Further differentially expressed transcription factors known to respond to P starvation were found in other co-expression modules. For example, *OsWRKY74* [40] and *AtWRKY75* are upregulated during P starvation and regulate P starvation induced genes, whereas AtWRKY6 and AtWRKY42 bind to the *PHO1* promoter under P sufficient conditions, inhibiting the transcription of PHO1 (PHOSPHATE1, involved in Pi xylem loading) [35]. Here, the poplar WRKY ortholog Potri.001G097200 (module “Brown”) was upregulated upon P starvation in both roots and leaves, whereas others (e.g. Potri.003G138600, Potri.006G263600, Potri.016G128300 from module “Blue”, Potri.001G044500 from module “Green”, Potri.003G169100, Potri.003G182200, Potri.014G096200, Potri.014G096200 from module “Pink”) were downregulated upon P starvation, especially in roots. Different expression patterns for different members of the large WRKY family suggested distinct functions for adaption to low P.

Ethylene-responsive element binding factors (ERFs) were especially abundant in the N- and C-related co-expression module. Upon P deprivation they were downregulated in roots and showed no or slight up-regulation in leaves indicating an influence of ethylene signaling on root responses. This speculation is supported by the observation that low P conditions in *Arabidopsis* integrate ethylene response with enhanced root hair formation and primary root growth arrest [36]. Furthermore, in *Arabidopsis* zinc finger proteins (ZATs) such as AtZAT6 have functions in root development and P acquisition [4,36]. Here poplar ZAT orthologs from module “Blue” (Potri.009G027700, Potri.001G235800, Potri.014G017300, Potri.001G295500, Potri.008G051200, Potri.002G119300) were responsive to P deprivation with a strong down-regulation in roots upon P starvation suggesting conserved functions of these genes in poplar and *Arabidopsis*.

An important result of our study was that the identified transcription factors were assigned to different hierarchical levels of the transcriptional network: those being involved in perception and signaling were present in top level (module “Green”), while others, which control long distance transport and morphological adjustments were found in secondary positions (modules “Brown” and “Blue”).

### Transcriptional regulation of P acquisition and P uptake

Under P deficiency the expression of genes for phosphate transporters (PHTs) and Purple Acid Phosphatases (PAPs) is upregulated [6–8]. Here, upregulated PAPs were present in the P-related module “Green” (Potri.005G233400, Potri.010G158200) but also in the secondary modules “Blue” (Potri.012G042200, Potri.015G031400, Potri.010G158200, Potri.005G233400, Potri.003G030700) and “Brown” (Potri.008G139100). PAPs can be secreted or have intracellular localization. The latter PAPs may contribute to increase the internal P reuse of the plant and are involved in leaf senescence due to their ability to form peroxides [41]. Here, most PAPs were found in module “Blue”, in which genes for stress responses and DNA/RNA remodeling clustered as major GO terms suggesting that these invoked enhanced P recycling.

An unexpected result was that none of the PHTs was assigned to module “Green”, although various PHTs respond strongly to P deficiency [7–9]. Here, *PtPHT1;12* and *PtPHT1;9* (Potri.001G318500, Potri.002G005500) were detected in module “Brown”, whose eigengenes were strongly correlated with biomass, Mg and S and in which GO terms for cell cycle, chromosome organization and DNA were enriched. The orthologs of PtPHT1;9 in *Arabidopsis*, AtPHT1;8 and AtPHT1;9, are crucial for P nutrition because they maintain high-affinity P uptake into the roots under P starvation [42]. Also the orthologs in rice, OsPHT1;9 and OsPHT1;10 function in P uptake [43]. Poplar *PtPHT1;12* and its *Arabidopsis* ortholog (*AtPHT1;5*) show enhanced expression under P deficiency and in senescing leaves [8,44][44]. Therefore, PtPHT1;12 may function in the redistribution of P inside the plant. Furthermore, in the module “Turquoise”, which forms a tertiary level in the co-expression network, further PHTs were upregulated. Module “Turquoise” was strongly negatively correlated with Fe and Mn, which suggests links between the latter elements and P translocation. Earlier studies support this suggestion because enhanced P uptake prevented over-accumulation and toxicity of Mn [45].

P release from plasma membranes replacing phospholipids by galactolipids is a common adaptation to P starvation [46,47]. In our study this response was evident at the transcriptome level. GO term enrichment indicated a correlation between membrane lipid remodeling and P concentrations in module “Green”. These results agree with studies in *Arabidopsis*, where at least parts of membrane lipid remodeling depend on P signaling and auxin/cytokinin crosstalk [48]. For example, the *Arabidopsis SQD1* (*SULFOQUINOVOSYLDIACYLGLYCEROL1*) as well as the rice ortholog are involved in sulfolipid synthesis and regulated by the phosphate content in plant tissues [49,50]. Here, a putative *SQD* (Potri.016G112600) was one of the most strongly upregulated genes in module “Green” and correlated with the P concentration supporting similar functions in the poplar and *Arabidopsis* P starvation response.

### Metabolism of mineral nutrients, sulfur, nitrogen and carbon upon P starvation

The concentrations of basic cations declined 1.5 to 2-fold (Ca, Mg, K) upon P starvation in poplar leaves, but did not fall below threshold values reported for sufficient nutrition in leaves of young *P*. × *canescens* (Ca: 15.9 mg g^-1^, Mg: 2 mg g^-1^, K: 7.6 mg g^-1^ [51]). This was also true for sulfur, whose foliar concentrations declined 3-fold under P deficiency (threshold: 3 mg g^-1^ [51]). Together these elements were positively correlated among each and also with the co-expression module “Turquoise” in which GO terms for sulfur metabolism and organic acid biosynthesis were enriched (S1 Fig). Production and exudation of organic acids are known responses to P deficiency because those metabolites can exchange anions such as Pi from soil particles, thereby increasing Pi availability [52]. The present data imply that the production of organic acids is not a direct response to declining cellular P concentrations but a down-stream consequence that may perhaps also require the perception of general changes in the cellular ion balance.

Cross talk exists between the P sensing and Zn as well as Fe nutrition involving the PHR1 gene regulation [53,54]. In our study increased Fe concentrations were accompanied by enhanced Al and Mn concentrations, whose over-accumulation may damage plant tissues [55] and correlated here with decreased tissue carbon concentrations. Furthermore, in our study the concentration of Zn and Fe were increased or relatively stable and significantly correlated with the eigengenes of module “Turquoise” (negative for Fe) and “Yellow” (positive for Zn, negative for Fe). In contrast to poplar, in *Arabidopsis* and rice the Zn concentrations are reduced or unaffected during P starvation, whereas the Fe concentrations increased considerably upon P starvation [11,56–60]. In *Arabidopsis*, Fe as well as P homeostasis are regulated via PHR1 that induces the iron storage protein FERRITIN1 [53]. Upon P starvation, iron is stored with ferritin in chloroplasts, which prevents precipitation with phosphate [57]. Our data indicate that chloroplastic metabolism must have been massively affected because module “Yellow” was enriched in the GO terms “photosynthesis”, “pigment biosynthesis”, “starch and carbohydrate metabolism” (S1 Fig). However, no significant up-regulation of ferritin was detected in leaves. In roots, one ferritin gene (Potri.016G124900) was induced, whereas two others (Potri.006G103900; Potri.010G184500) were decreased (S1 Table). In agreement with these observations we also detected up-regulation of an iron transport protein (Potri.015G117900) in roots, whose *Arabidopsis* orthologs were expressed highly in the outer layers of the roots under Fe-deficiency [61]. In contrast, a putative vacuolar iron transporter (Potri.010G104200) showed decreased expression in poplar roots. These observations suggest that P deficiency modifies iron uptake and distribution in roots, probably similar to that in *Arabidopsis* [57]. How different species deal with Fe under P starvation may also depend on their ability to produce organic acids which can sequester cations. For example, in strawberry roots the Fe concentration is strongly reduced upon P starvation [62]. Both P and Fe starvation lead to changes in root carboxylate metabolism and an increased exudation of citrate [62]. This suggests similar pathways for P and Fe signaling and independent regulation of Fe metabolism during P starvation in different plants.

In poplar, carbohydrates accumulated in roots upon P starvation [7] in agreement with other studies [63] but the total tissue carbon concentrations were not significantly affected (this study). One reason for this observation could be a shift in metabolic use of carbon-bearing compounds. For example, in P starved poplars, we found a very high up-regulation of PPC (phosphoenolpyruvatcarboxylase) and the PPC activating kinase PPCK. PPC catalyzes the production of oxaloacetate from phosphoenolpyruvate and bicarbonate releasing Pi. Oxaloacetate can then be secreted or further metabolized into other organic acids and used for energy production.

Furthermore, P starvation has consequences for N nutrition. Both carbon and nitrogen clustered with module “Blue”, which is characterized by defense responses including respiratory burst (ethylene, phosphorelay signal transduction system) and histone modification suggesting massive transcriptional reprogramming. Unlike most of the mineral nutrients, tissue N concentrations increased, which may happen when growth is inhibited but N uptake continues. Here, we noted a strong induction of *BSP* (Bark Storage Protein) gene expression. In poplar, *BSP* expression increases in fall resulting in BSP protein accumulation and N storage during winter [64,65]. Our result suggests that the strong growth reduction imposed by P deficiency led to N accumulation and as a further consequence induced the formation of BSPs to store N. This speculation is supported by earlier results showing that shoot growth influenced BSP promoter activity [66]. Seasonal N cycling in perennial species also involves protein degradation [67]. BSP induction may, thus, be related to protein degradation, which was found here among the directly P correlated transcriptional responses.

### Conclusions

P is often the least available nutrient in soil. Here, we demonstrate that P starvation leads not only to P depletion in the tissues but also to massive modifications in the concentrations of other nutrient elements. Under these conditions, in roots as well as in leaves several 1000 genes were differentially expressed. Based on this finding we conclude that a massive acclimation of poplar metabolism is required to cope with this critical situation. A central goal of this study was to find out whether it was possible to separate DEGs related to P starvation and those responding to down-stream events. We successfully identified a hierarchical network structure in which co-expressed genes clustered in modules that correlated with distinct nutrient element concentrations in roots and leaves. The genes in the primary module which comprised only a small fraction (about 5%) of all DEGs highlighted a tight connection between tissue P concentrations and P recycling due to membrane and nucleotide remodeling. The co-expression of genes for metabolic adaptation to P starvation with master switches for the regulation of P uptake such as SPX proposes cross-talk between these processes. Our results further imply similar regulation of these processes in roots and leaves because with few exceptions the DEGs in this module showed the same P starvation response for different tissues. We therefore conclude that the primary module contained mainly those genes, whose transcript abundance is regulated by the tissue P availability.

Surprisingly, PHTs were not present in the P correlated module but occurred in down-stream modules. DEGs that represent other typical P starvation responses such as the production of organic acids were neither directly correlated with P, but occurred in a down-stream module that was correlated with sulfur. We, thus, infer that under prolonged conditions of P starvation acclimation of tissue metabolism to P deficiency is the primary requirement and acclimation of P acquisition a down-stream response.

We found correlative links between N enhancement, BSP up-regulation, and growth depression for P starved poplars. Since BSPs are storage proteins with strong seasonal regulation, our findings also highlight that P starvation can interfere with tree-specific physiological processes. Overall, the modular transcriptional responses to P starvation uncovered new links between nutrients and the molecular regulation of biological functions that can be exploited for a better understanding of the plant nutrient balance.

## Supporting information

S1 Fig Enriched GO terms in all modules visualized by REVIGO. (PPTX)

S1 Table Annotation, fold-changes and module assignment of differentially expressed genes in response to P starvation. (XLSX)

S2 Table Overview on enriched GO terms in all modules. (XLSX)

S3 Table List of most strongly differentially expressed genes in module “Green”, “Brown” and “Blue” shown in Fig 6, abbreviations of gene names and comparison with a core P responsive gene set identified in P starved *Arabidopsis* [12]. (XLSX)
