# Supplementary material for: Dissecting nutrient-related co-expression networks in phosphate starved poplars
Source: PLoS One. 2017 Feb 21;12(2):e0171958. doi: 10.1371/journal.pone.0171958 (PMC5319788; doi:10.1371/journal.pone.0171958)
Supplement: S1 Fig — (PPTX) [file pone.0171958.s001.pptx]

## Slide 1
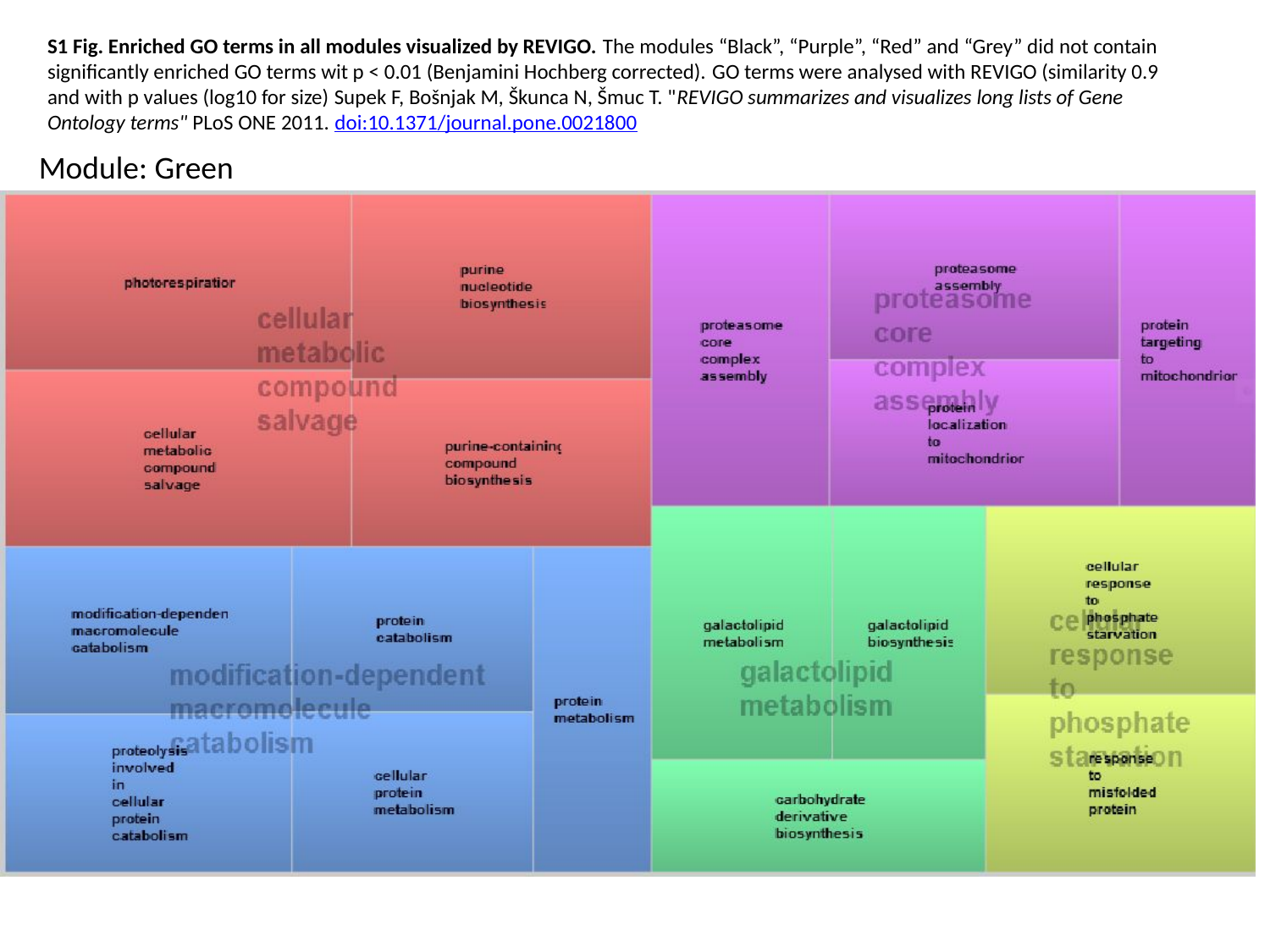

S1 Fig. Enriched GO terms in all modules visualized by REVIGO. The modules “Black”, “Purple”, “Red” and “Grey” did not contain significantly enriched GO terms wit p < 0.01 (Benjamini Hochberg corrected). GO terms were analysed with REVIGO (similarity 0.9 and with p values (log10 for size) Supek F, Bošnjak M, Škunca N, Šmuc T. "REVIGO summarizes and visualizes long lists of Gene Ontology terms" PLoS ONE 2011. doi:10.1371/journal.pone.0021800
Module: Green

## Slide 2
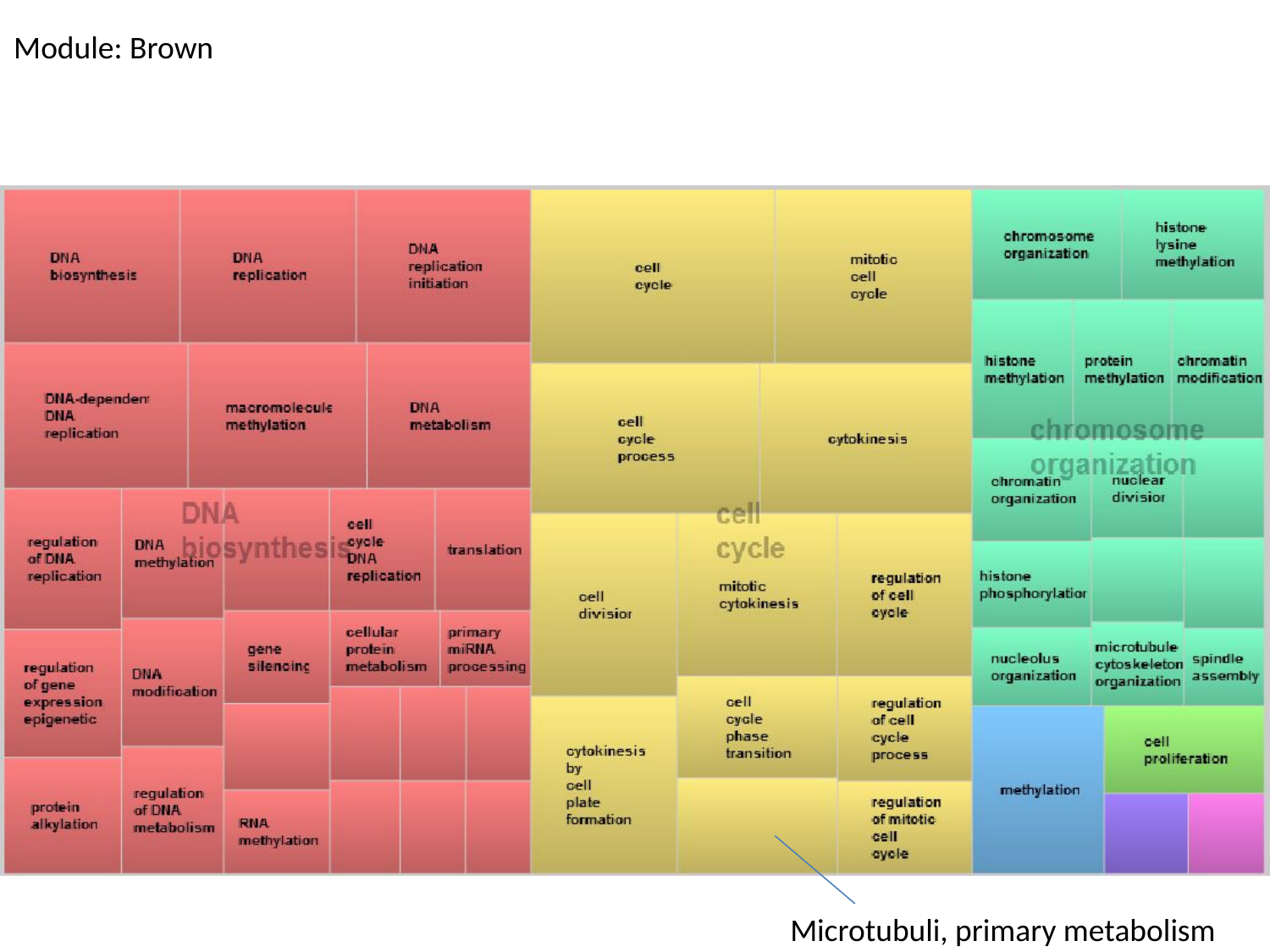

Module: Brown
Microtubuli, primary metabolism

## Slide 3
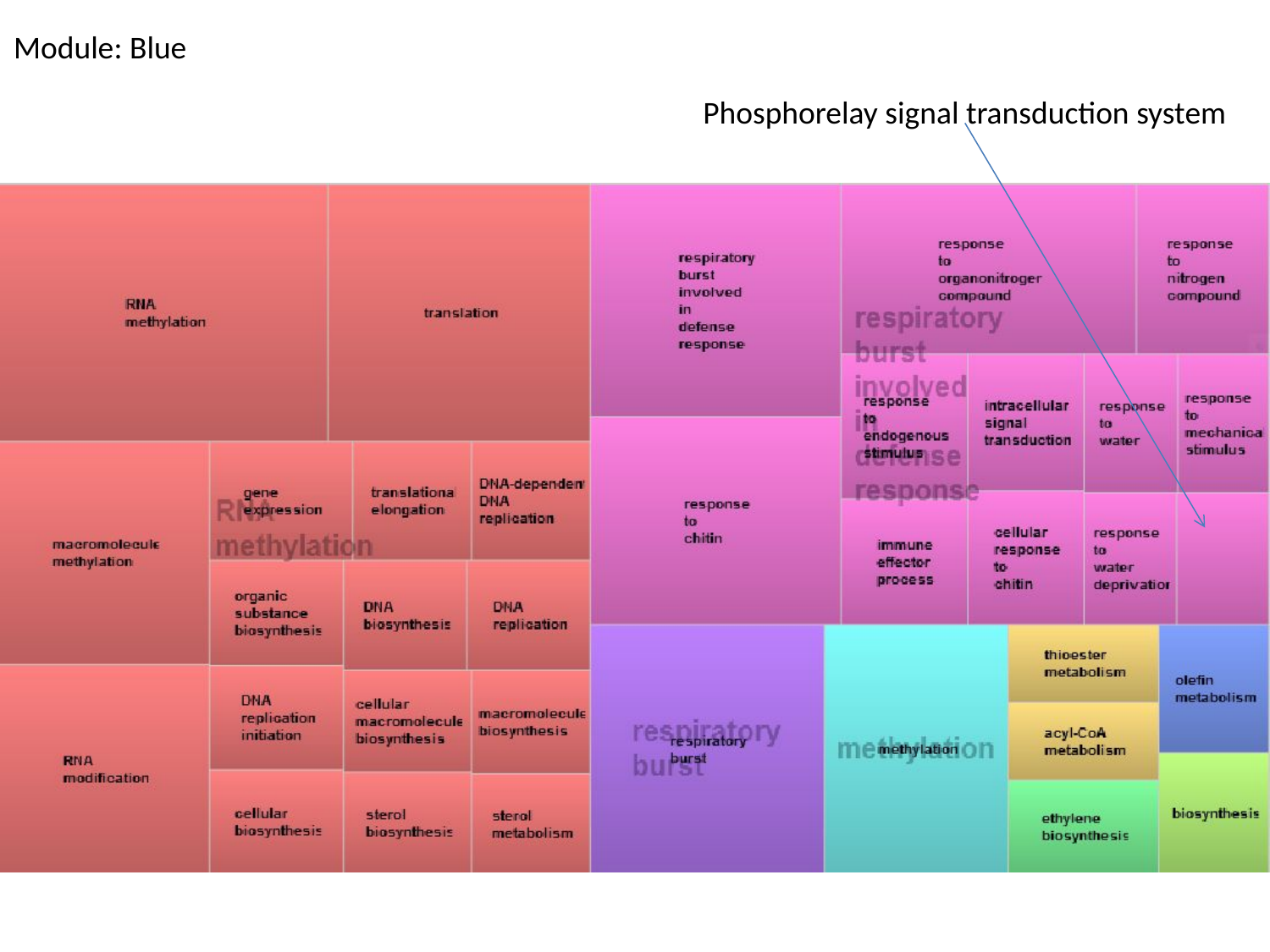

Module: Blue
Phosphorelay signal transduction system

## Slide 4
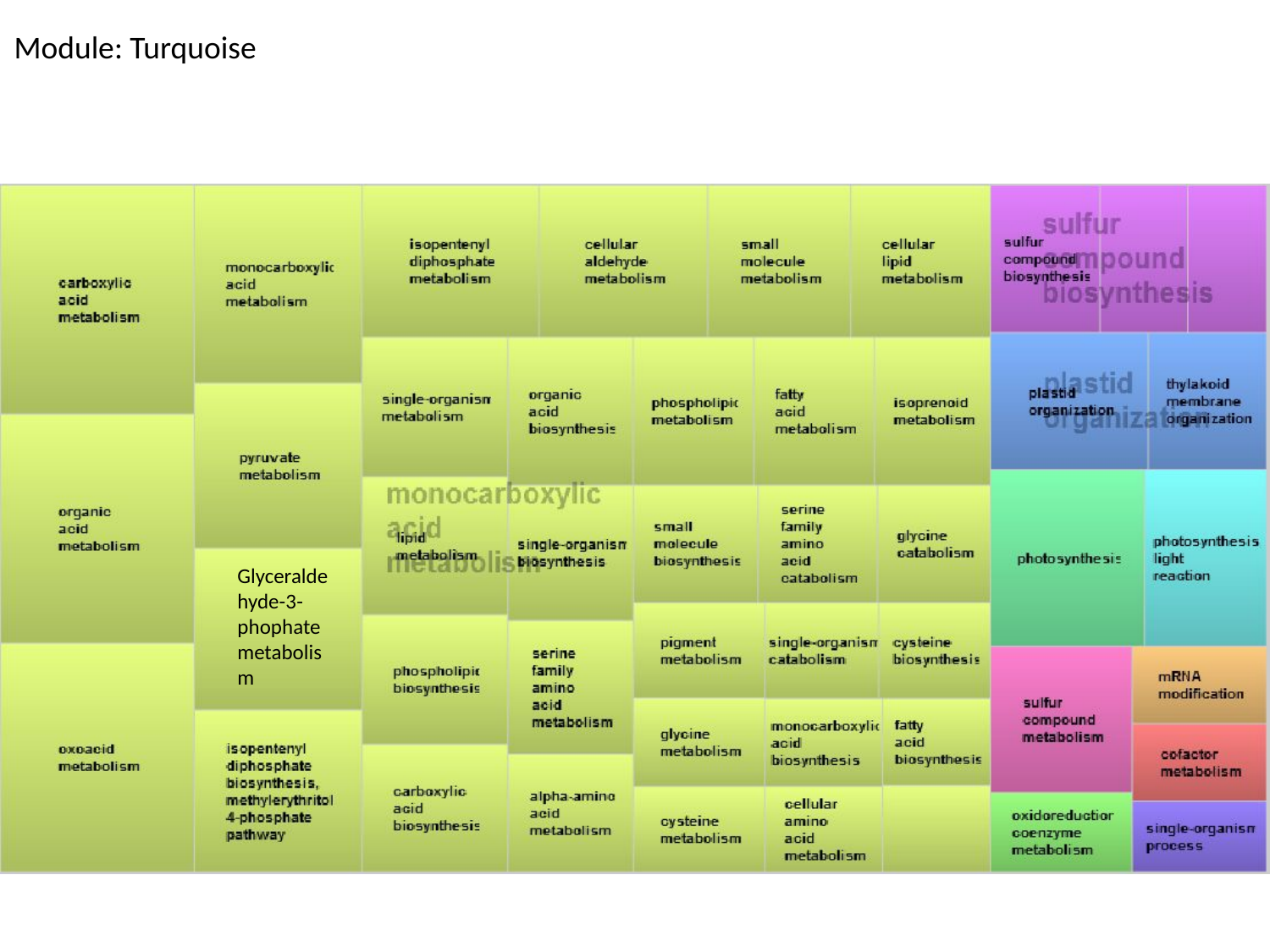

Module: Turquoise
Glyceraldehyde-3-phophate metabolism

## Slide 5
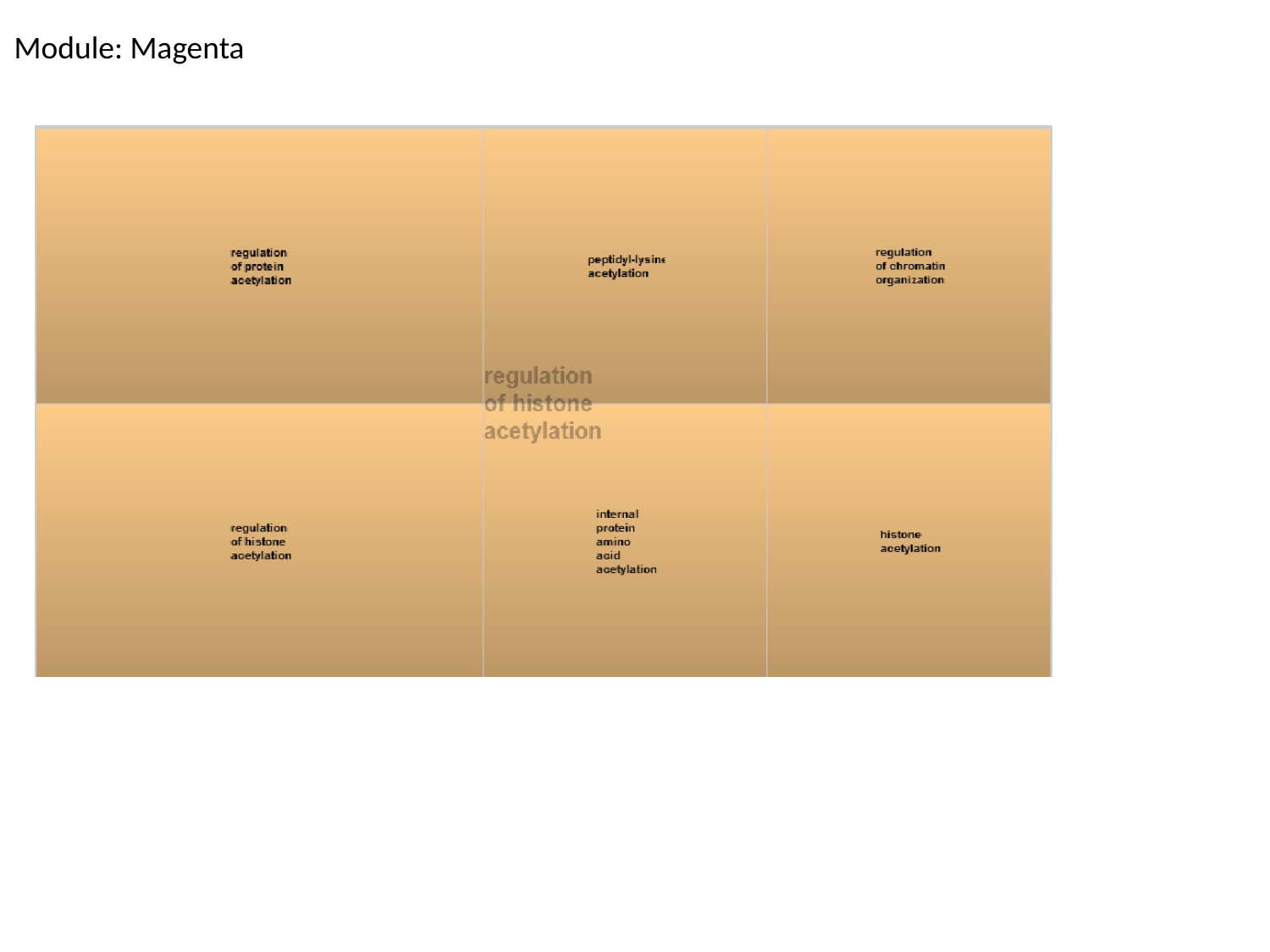

Module: Magenta

## Slide 6
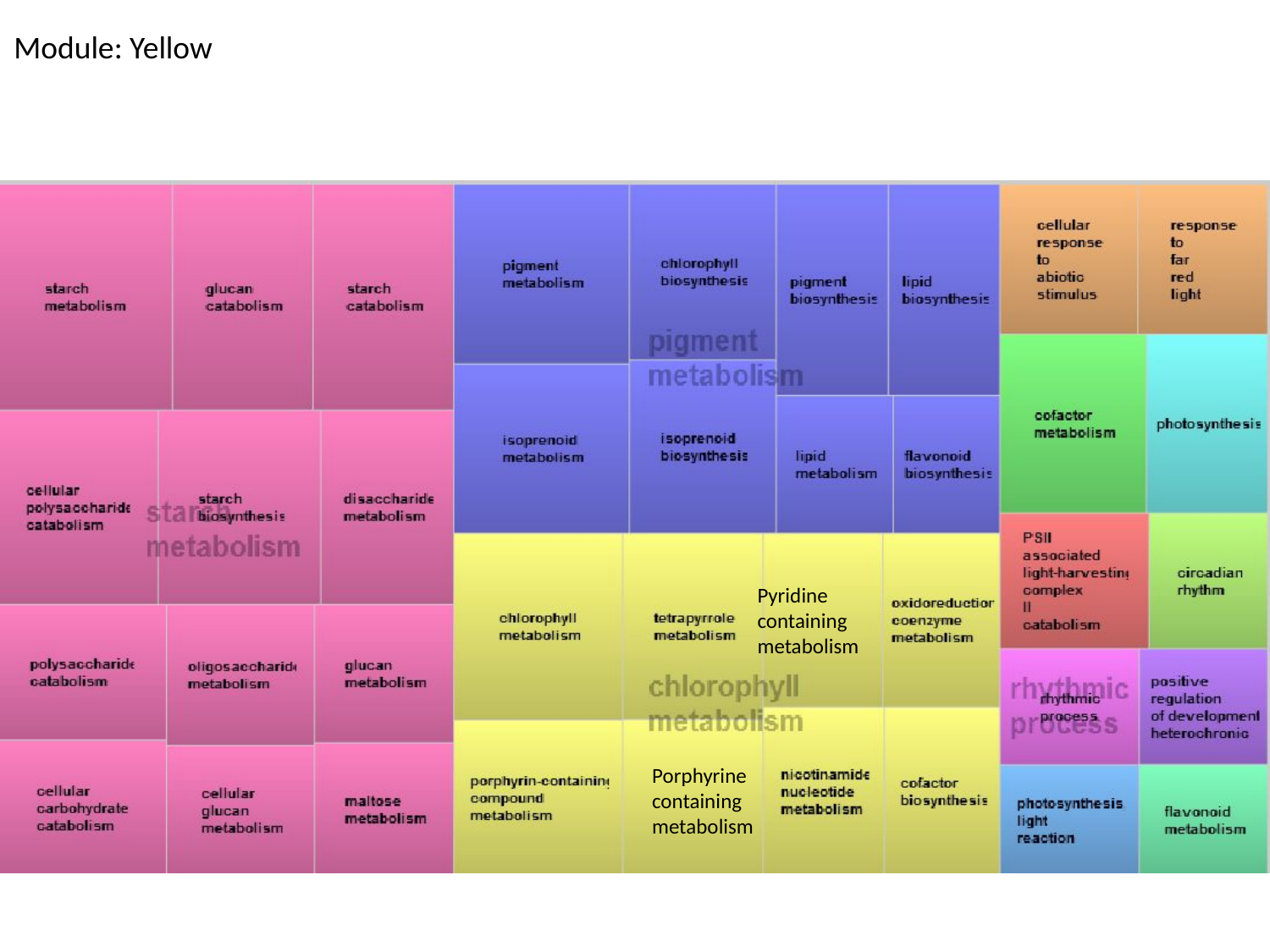

Module: Yellow
Pyridine containing metabolism
Porphyrine containing metabolism

## Slide 7
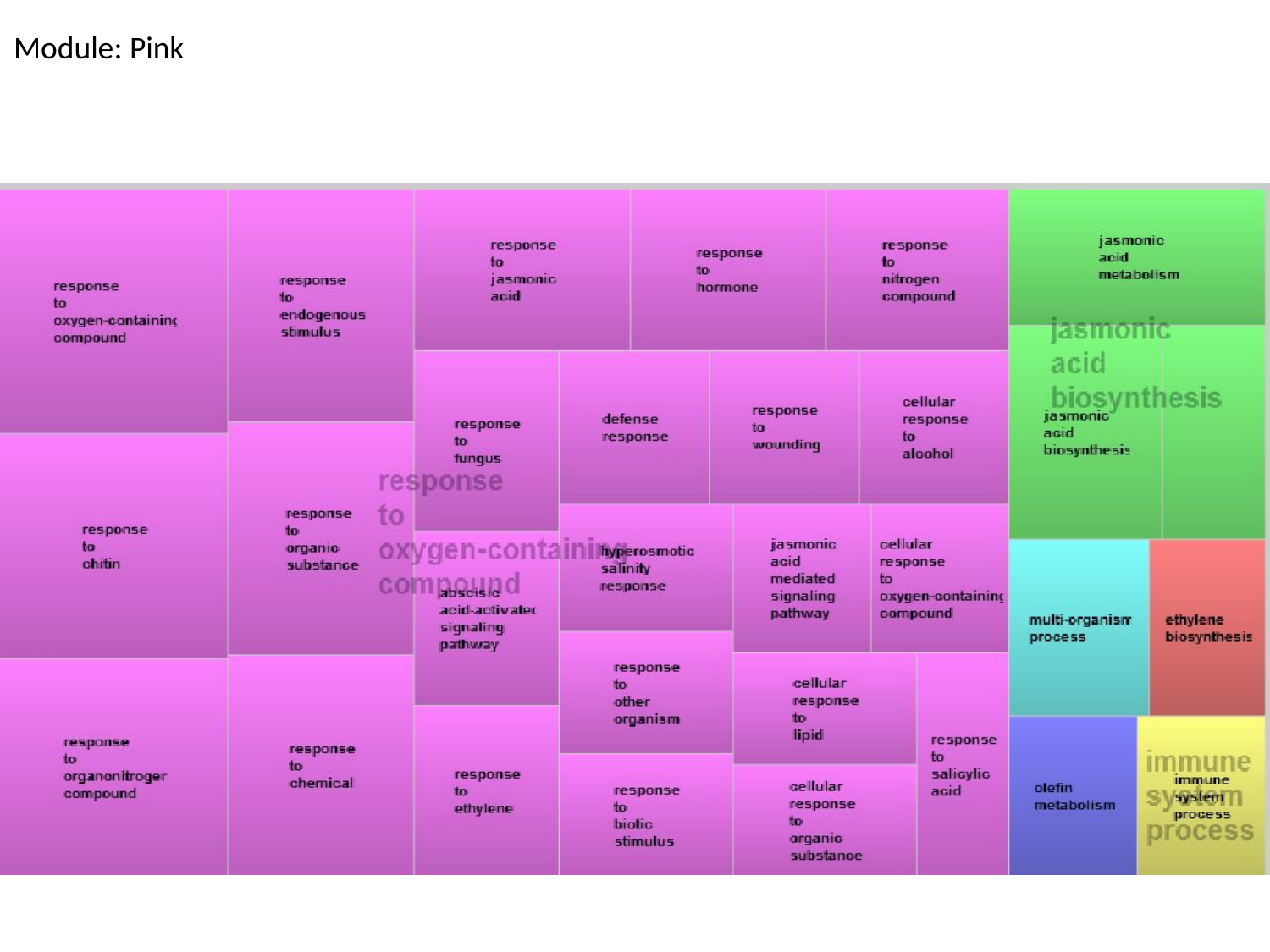

Module: Pink
